# Pegbelfermin for reducing transaminase levels in patients with non-alcoholic steatohepatitis: a dose-response meta-analysis of randomized controlled trials

**DOI:** 10.3389/fmed.2024.1293336

**Published:** 2024-04-05

**Authors:** Yangguang Lu, Bohuai Yu, Yiran Bu, Jialing Lou, Yan Jin

**Affiliations:** ^1^The First School of Medicine, School of Information and Engineering, Wenzhou Medical University, Wenzhou, China; ^2^Department of Infectious Diseases, Tongde Hospital of Zhejiang Province, Hangzhou, China

**Keywords:** non-alcoholic steatohepatitis, Pegbelfermin, BMS-986036, fibroblast growth factor 21, transaminase reduction, dose-response meta-analysis

## Abstract

**Background:**

The efficacy of Pegbelfermin (PGBF) in treating non-alcoholic steatohepatitis (NASH) remains controversial. Therefore, we conducted a dose-response meta-analysis to explore the effect and pattern of PGBF at different dosages and treatment durations on transaminase reduction in NASH patients.

**Methods:**

We conducted searches on PubMed, Embase, Cochrane Library, Web of Science, and ClinicalTrials.gov, and supplemented the search with gray literature and manual searches. Randomized controlled trials (RCTs) evaluating the efficacy of PGBF in NASH patients were included. Risk of bias was assessed by Cochrane Risk of Bias Tool 2.0. We used random-effects models, generalized least squares regression, constrained maximum likelihood, and restricted cubic splines to explore the dose-response relationship. Egger's linear regression was employed to assess publication bias. The study is registered with PROSPERO, CRD42023448024.

**Results:**

Four RCT studies from the period 2018–2023, involving 546 participants, were included. No participants discontinued PGBF treatment due to adverse events. High-dose PGBF treatment significantly reduced transaminase levels in NASH patients compared to the low-dose group (ALT %: MD = 14.94, 95% CI = 2.11–27.77; AST %: MD = 9.05, 95% CI = 3.17–14.92). Longer treatment duration further decreased transaminase levels (ALT%: MD = 8.81, 95% CI = 4.07–13.56; AST%: MD = 6.72, 95% CI = 2.62–10.81). Egger's test did not reveal significant publication bias (*p* > 0.05). Further investigation indicated a ceiling effect of PGBF dosage on transaminase reduction at 30 mg/week, and NASH patients experienced a rebound in transaminase levels after 28 weeks of continuous treatment.

**Conclusion:**

There is a positive correlation between PGBF dosage and transaminase reduction within a certain range, showing an overall non-linear dose-response relationship. This finding provides guidance for the clinical application of PGBF. Clinicians should be mindful of the dosage ceiling at 30 mg/week and monitor changes in transaminase levels after 28 weeks for timely adjustments in PGBF dosage.

**Systematic review registration:**

PROSPERO, CRD42023448024. https://www.crd.york.ac.uk/PROSPERO/display_record.php?RecordID=448024

## 1 Introduction

Non-alcoholic steatohepatitis (NASH) is a progressive form of non-alcoholic fatty liver disease characterized by hepatic steatosis, inflammation, and hepatocellular injury (1), which may lead to liver fibrosis (2). A considerable proportion of NASH patients may progress to cirrhosis, hepatocellular carcinoma, liver failure, or even death (3). A global meta-analysis revealed that the prevalence of NASH in adults worldwide ranges from 1.5 to 6.5% (4), and it is on the rise, partly due to the increasing incidence of type 2 diabetes (5). However, there are currently no approved drugs for NASH treatment (6), and lifestyle interventions like diet and exercise for weight loss remain the standard therapeutic measures. While weight reduction can partially improve liver function (1), the increasing prevalence of obesity suggests the difficulty in maintaining weight loss (7). Therefore, NASH imposes significant clinical, economic, and societal burdens (8). Given the inadequacy of current NASH treatment strategies, there is an urgent need to develop a drug targeting this condition.

Fibroblast Growth Factor 21 (FGF21) is a non-mitogenic hormone that regulates glucose and lipid metabolism (9) and plays a critical role in energy homeostasis (10). Primarily secreted by the liver, FGF21 enhances fatty acid metabolism (11). Endogenous FGF21 has a short half-life of 1–2 h, but various modification strategies have been employed to create longer-acting FGF21 analogs (12). Pegbelfermin (PGBF) is a polyethylene glycol-conjugated recombinant analog of human FGF21, with an extended half-life that allows for weekly dosing (13). Preclinical studies using NASH mouse models have demonstrated that PGBF effectively improves liver histology (14). Therefore, the positive effects of FGF21 and its engineered analog PGBF on lipid metabolism and liver function suggest their potential as therapeutic agents for NASH (15). However, despite most current studies reporting favorable therapeutic outcomes and only mild adverse reactions with PGBF in NASH, controversies persist regarding its specific effects, optimal dosage, and suitable dosing frequency (13, 14). Consequently, it is essential to further investigate the effectiveness and mode of action of PGBF in NASH treatment.

Transaminases, including alanine transaminase (ALT) and aspartate transaminase (AST), are general markers of hepatocellular injury (16). Elevated transaminase levels are closely associated with an increased risk of developing end-stage liver disease, making them important prognostic factors for NASH patients (17). Furthermore, reductions in transaminase levels have been linked to improvements in hepatic steatosis and inflammation observed in patients' liver biopsies, although the correlation between transaminase reduction and improvement in liver histological status may not necessarily imply a causal relationship (18, 19). Thus, studying the effect of PGBF on transaminase reduction can serve as an indicator of its therapeutic efficacy for NASH.

Considering the continuous increase in the global prevalence of NASH and the ongoing debate surrounding the efficacy of PGBF treatment, further exploration of its clinical application and effects is of utmost importance. Currently, there is no systematic review or meta-analysis regarding the correlation between PGBF and its therapeutic effects on NASH. Therefore, we conducted a dose-response meta-analysis to investigate the effect and pattern of transaminase reduction in NASH patients treated with different doses and durations of PGBF. By understanding the specific therapeutic effects of PGBF in various modes of administration for NASH, this analysis aims to provide guidance to clinicians for the application of PGBF and the development of relevant guidelines.

## 2 Materials and methods

### 2.1 Search strategy

This systematic review and meta-analysis were conducted following the Preferred Reporting Items for Systematic Reviews and Meta-Analyses (PRISMA) guidelines (20) and were based on a pre-designed scheme (PROSPERO protocol: CRD42023448024). As all analyses used publicly available aggregated data and not individual data, no institutional review board ethical approval or patient informed consent was required.

Two researchers systematically searched studies published up to July 18, 2023, in the PubMed, Embase, Cochrane Library, and Web of Science databases. Additionally, completed but unpublished trials were searched on the ClinicalTrials.gov website. The language was restricted to English. The search utilized a Boolean combination of keywords “NASH” and “PGBF,” along with a combination of subject headings and free-text terms. Medical Subject Heading (MeSH) terms were applied for PubMed and Cochrane Library searches, while Embase was searched using Emtree terms. Furthermore, gray literature was searched from WorldCat, Google Scholar, and Open Gray, and reference lists of any systematic or narrative reviews found during the search for potentially relevant studies were screened. Finally, a search was conducted in PROSPERO to ensure there were no similar ongoing studies.

### 2.2 Selection criteria

Articles meeting the following criteria were included: (1) Reporting randomized controlled trials (RCTs) evaluating the effectiveness of PGBF in treating human NASH patients; (2) Studies including at least three dose levels; (3) Reporting the mean change and standard deviation (SD) of ALT and AST levels at different time points during the treatment compared to baseline for each group. Studies with the following characteristics were excluded: (1) Follow-up duration of <16 weeks; (2) Studies with fewer than 50 participants; (3) Unclear diagnosis of NASH or description of the intervention. In cases of multiple publications from the same trial, the publication with the longest follow-up period was included. Two researchers independently selected the studies, and any discrepancies were resolved through arbitration by a third author.

### 2.3 Data extraction

After removing duplicate studies from the database search, two researchers independently assessed potentially eligible studies. Initial screening was done by reading the titles and abstracts of the publications. After the initial screening, both researchers read the full texts of each publication and reevaluated their eligibility. Relevant data, including title, first author's name, publication year, country or region of participants, sample size, demographic information, baseline data of transaminase, dosage information of different intervention groups, follow-up time, and outcome data at different time points under different doses, were extracted from all eligible studies. A data extraction table was designed to record the results of data extraction for included studies. WebPlotDigitizer 4.6 software (https://automeris.io/WebPlotDigitizer/, accessed on July 23; (21)) was used for data extraction from images (22). Any discrepancies arising during data extraction were resolved through consensus.

Based on the experimental design of the included studies, we conducted quality assessment using the revised Cochrane Risk of Bias Tool (ROB) 2.0. ROB is a standard scale used in meta-analyses to evaluate the quality of RCT studies (23). The overall risk of bias was assessed as low, some concerns, or high. If at least four criteria were assessed as “low,” and no criteria were assessed as “high,” the overall bias risk was considered to be relatively low. Two reviewers independently performed all quality assessments, and any discrepancies were resolved through arbitration by a third author.

### 2.4 Statistical analyses

In this meta-analysis, the mean change and its SD of transaminase levels compared to baseline were considered as effect sizes. The mean change was expressed as a percentage. We calculated the average weekly dose of PGBF for each dosage group and selected “10 mg/week” and “20 mg/week” as the two standard dosage groups for subsequent comparisons. The reason for considering these doses as standard is that, among the included studies, these two dosage levels of PGBF were most frequently reported. If an included study did not have a standard dosage group, we selected the experimental group with the PGBF dose closest to the standard dosage group (difference <3 mg) and treated it as the standard dosage group. Furthermore, the “average weekly PGBF dose” and “treatment duration” were considered as “dose” in the dose-response meta-analysis.

To observe whether high doses or longer durations of PGBF result in superior therapeutic effects, we employed a random-effects model to aggregate the mean differences (MD) of transaminase changes corresponding to the highest and lowest “doses” in different studies and calculated the 95% confidence interval (CI). Cochrane's *Q*-test and *I*^2^ statistic were used to assess heterogeneity among studies, where *I*^2^ exceeding 50% and 75% were considered moderate and high heterogeneity, respectively (24). Egger's linear regression test was used to assess publication bias (25). In order to examine whether the superior therapeutic effects of high doses or longer durations of PGBF were due to bias or errors from a single study, we conducted a sensitivity analysis using a “leave-one-out” approach to further elucidate the impact of PGBF on transaminase reduction in NASH patients. Next, we explored the non-linear trend between PGBF dose and treatment duration with transaminase changes using a restricted cubic spline model (26), applying a generalized least squares regression at predefined percentiles (10th, 50th, and 90th), and then summarizing specific estimates using constrained maximum likelihood (27). Additionally, we calculated the *p*-value for non-linearity, and for linear relationships, we used a general linear equation for pooling (27, 28).

All statistical analyses were performed using R 4.2.3 software package (Ross Ihaka, Robert Gentleman), and a *p*-value <0.05 was considered statistically significant.

## 3 Results

### 3.1 Study characteristics

**Figure 1:**
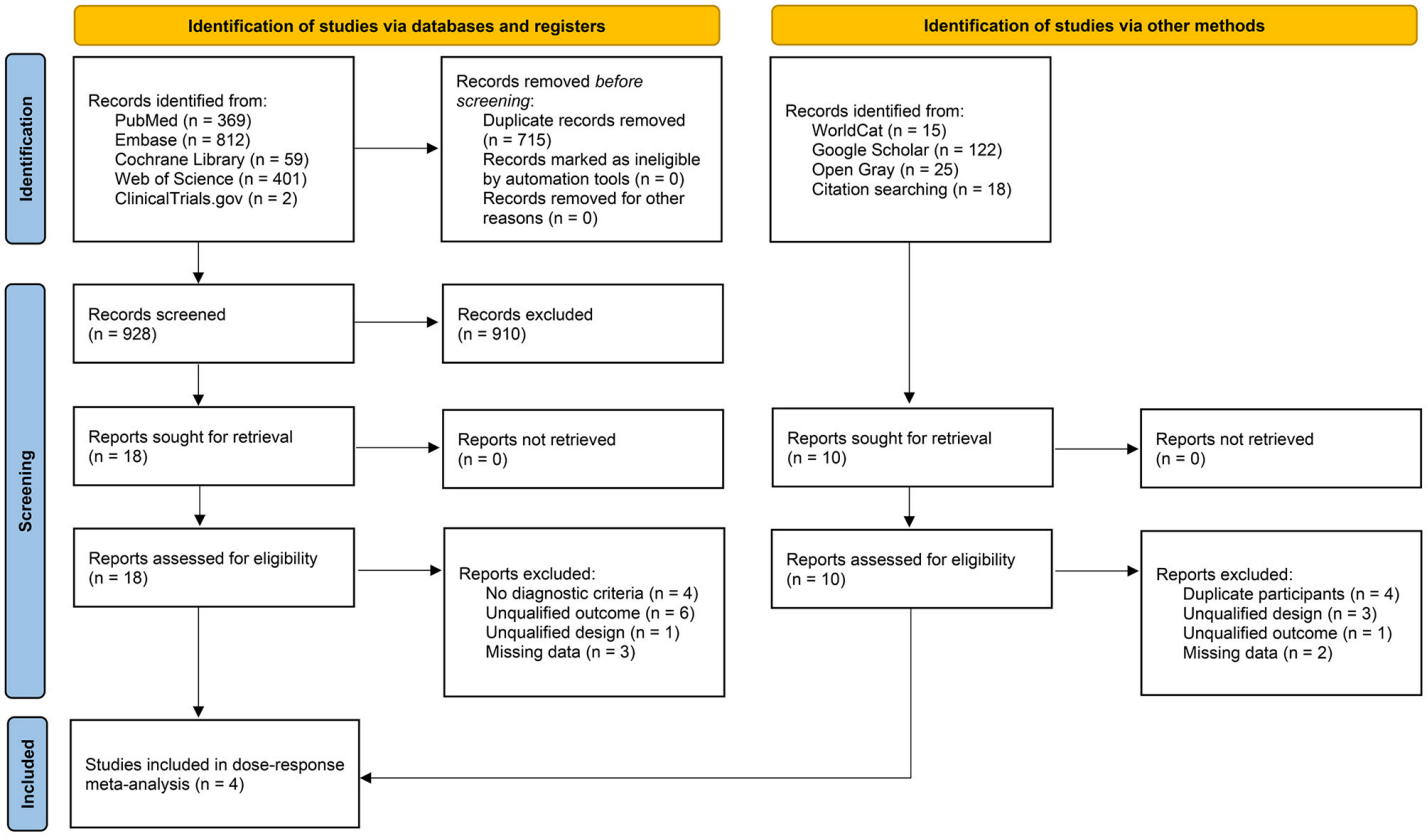
PRISMA flow diagram including reasons for exclusion of full-text articles.

We identified a total of 1,823 publications through the search process, out of which 1,819 publications were excluded, and four studies were finally included (Figure 1) (13, 14, 29, 30). We summarized the relevant information and characteristics of the included studies in Table 1. These four studies were published between 2018 and 2023 and involved a total of 546 participants. All participants remained on the treatment without discontinuation due to adverse events or treatment-related severe adverse events, with some studies reporting a few minor adverse reactions such as diarrhea (14). All studies were conducted in the United States. Among them, two studies simultaneously included participants from both the United States and Japan (29, 30), while one study included participants from both the United States and Canada (13). Although the follow-up time and data collection points were not consistent across all studies, all studies reported data at weeks 4, 8, and 12. All studies identified the “20 mg/week” standard dosage group, but the study conducted by Sanyal et al. (14) in 2018 did not report the “10 mg/week” standard dosage group.

**Table 1:** Characteristics of the four included studies.

**References**	**Year**	***N***	**Age (mean ± SD)**	**Male (%)**	**Participants' region**	**Follow-up time**	**Baseline information (mean** ± **SD)**		**Group design**
							**ALT**	**AST**	
Abdelmalek et al. (29)	2023	154	59.4 ± 8.8	36.4	US and Japan	48 weeks	48.6 ± 26.3	46.0 ± 25.2	Placebo, 10 mg QW, 20 mg QW, 40 mg QW
Charles et al. (13)	2019	120	56.0 ± 10.0	55.0	US and Canada	18 weeks	30.0 ± 17.0	24.0 ± 12.0	Placebo, 1 mg QD, 5 mg QD, 20 mg QD, 20 mg QW
Loomba et al. (30)	2023	197	56.9 ± 9.5	41.1	US and Japan	48 weeks	53.4 ± 32.9	45.7 ± 22.7	Placebo, 10 mg QW, 20 mg QW, 40 mg QW
Sanyal et al. (14)	2018	75	50.3 ± 11.4	35.8	US	16 weeks	72.1 ± 41.2	52.7 ± 33.9	Placebo, 10 mg QD, 20 mg QW

N, number of participants; SD, standard difference; QW, quaque week; QD, quaque day.

After evaluating using the ROB 2.0 tool, we assessed the risk of bias for these four studies (Table 2). Only two studies were rated as having “some concerns” in the “Missing outcome data” domain due to substantial loss to follow-up during the follow-up period (13, 14). Nevertheless, all four studies were considered to have a low risk of bias.

**Table 2:** Risk of bias assessment with Cochrane ROB tool 2.0.

**Evaluation items**	**Abdelmalek et al. (29)**	**Charles et al. (13)**	**Loomba et al. (30)**	**Sanyal et al. (14)**
Randomization process	Low	Low	Low	Low
Deviations from intended interventions	Low	Low	Low	Low
Missing outcome data	Low	Some concerns	Low	Some concerns
Measurement of the outcome	Low	Low	Low	Low
Selection of reported results	Low	Low	Low	Low
Overall bias	Low	Low	Low	Low

### 3.2 Dose of PGBF and treatment time on reducing transaminase

**Figure 2:**
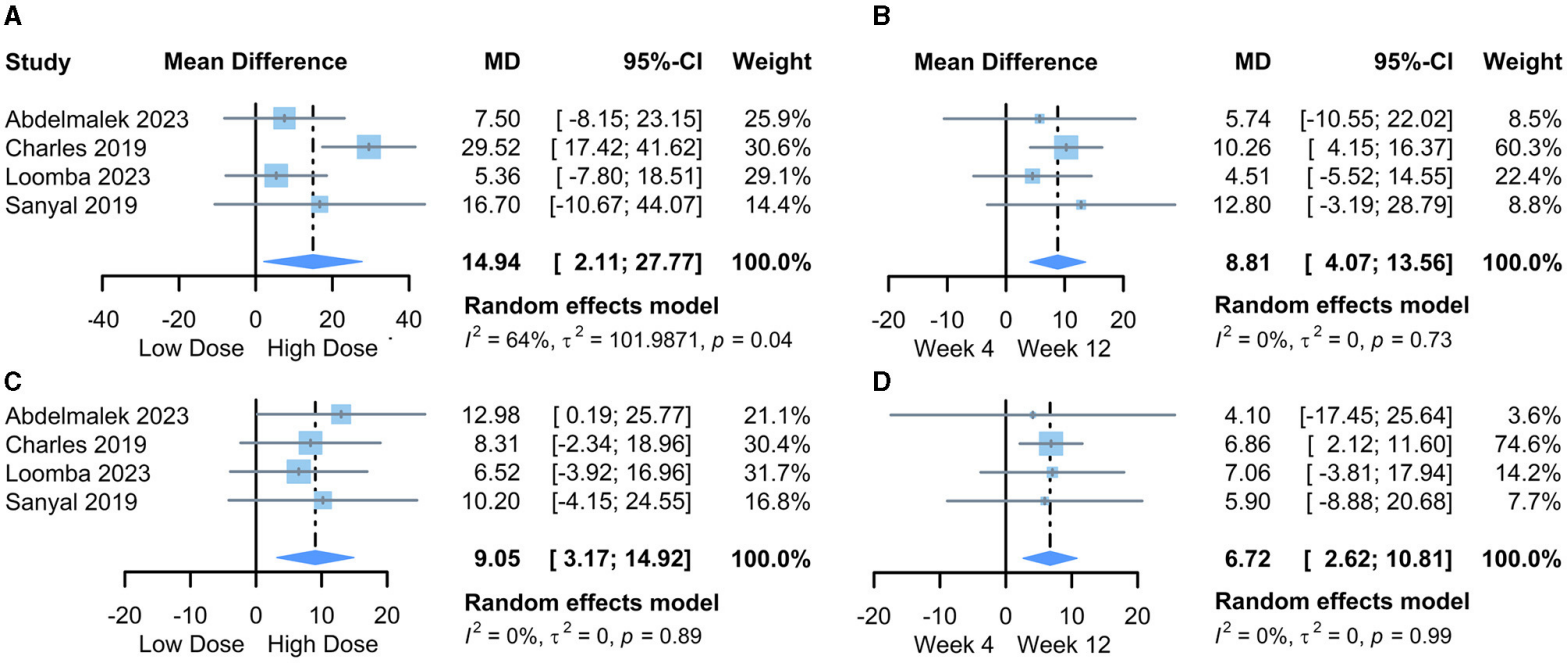
Forest plot of the meta-analysis between dose or time point groups. **(A)** The difference in the amount of ALT change, lowest dose vs. highest dose; **(B)** The difference in the amount of ALT change, week 4 vs. week 12; **(C)** The difference in the amount of AST change, lowest dose vs. highest dose; **(D)** The difference in the amount of AST change, week 4 vs. week 12. MD, mean difference; CI, confidence interval.

We first pooled the mean differences (MD) of transaminase changes compared to baseline for the lowest and highest PGBF dosage groups at week 12 in each study. The results from the random-effects model indicated that high-dose PGBF treatment was more effective in reducing ALT (Figure 2A) and AST (Figure 2C) levels in NASH patients compared to the low-dose group (ALT %: MD = 14.94, 95% CI = 2.11–27.77, *I*^2^ = 64%; AST %: MD = 9.05, 95% CI = 3.17–14.92, *I*^2^ = 0%). Egger's linear regression showed no significant publication bias (ALT: *p* = 0.7855; AST: *p* = 0.2851).

Next, we pooled the MD values of transaminase changes compared to baseline for the “20 mg/week” standard dosage group at weeks 4 and 12 in each study. The results from the random-effects model indicated further reductions in ALT (Figure 2B) and AST (Figure 2D) levels at week 12 compared to week 4 in PGBF-treated patients (ALT %: MD = 8.81, 95% CI = 4.07–13.56, *I*^2^ = 0%; AST %: MD = 6.72, 95% CI = 2.62–10.81, *I*^2^ = 0%). Egger's linear regression showed no significant publication bias (ALT: *p* = 0.7124; AST: *p* = 0.4895).

The results of sensitivity analysis showed a slight reduction in the significance of the difference in transaminase changes after excluding data from the study by Charles et al. (13). However, the trend of higher-dose PGBF treatment and longer treatment time showing favorable effects in reducing transaminase levels persisted. Therefore, further exploration of the dose-response relationship between PGBF dosage and treatment time on transaminase reduction is necessary.

### 3.3 Dose–response relationships related to ALT

**Figure 3:**
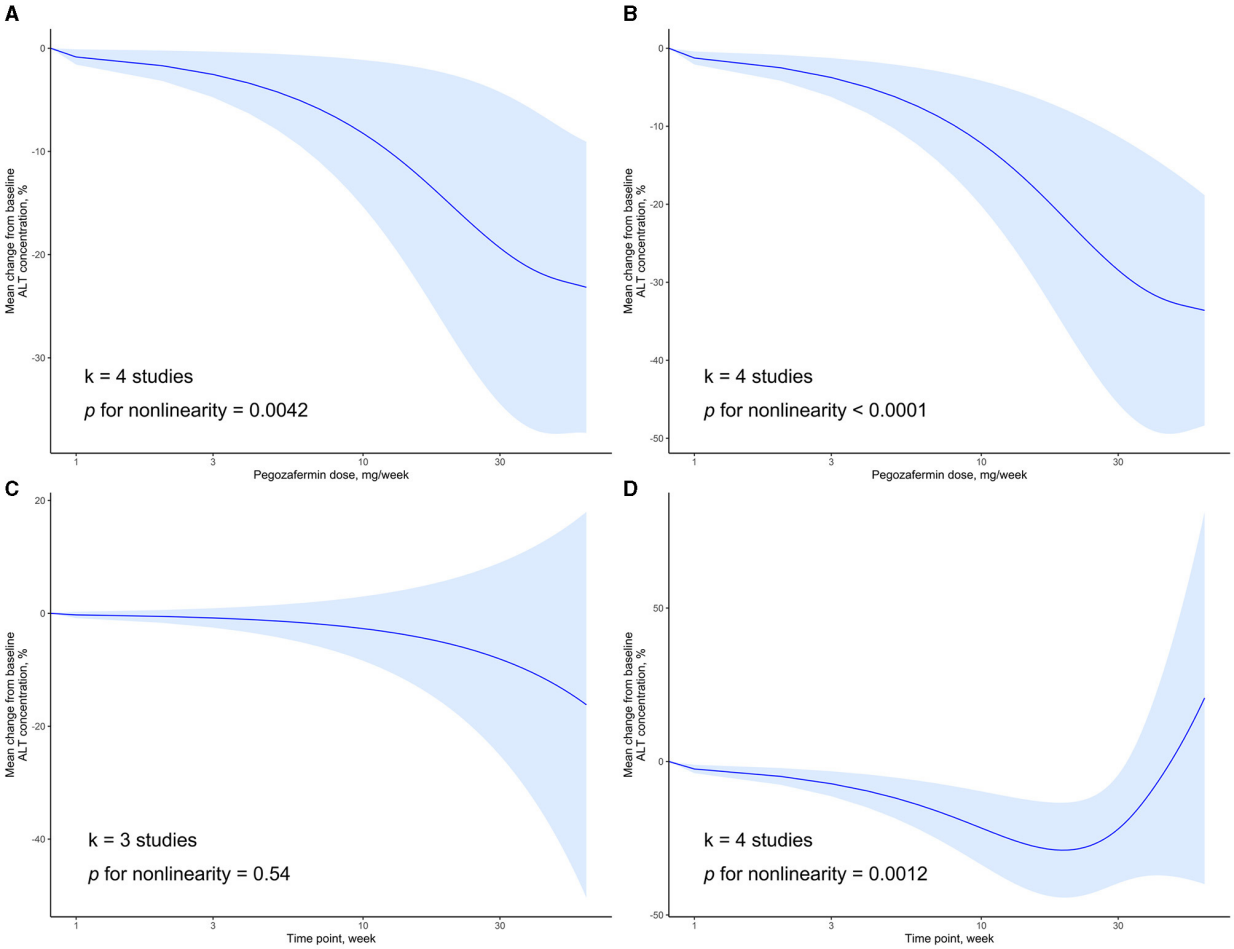
Dose-response relationship of ALT change to time point or PGBF dose. **(A)** The nonlinear relationship between ALT change and dose at week 4; **(B)** The nonlinear relationship between ALT change and dose at week 12; **(C)** The linear relationship between ALT change and time point at a dose of 10 mg/week; **(D)** The nonlinear relationship between ALT change and time point at a dose of 20 mg/week.

We examined the dose-response relationship between PGBF dosage, treatment time, and changes in ALT levels. At week 4, there was a non-linear relationship between PGBF dosage and ALT changes (*p* = 0.0042). As the PGBF dosage increased, the effect of ALT reduction increased, with the most significant impact observed in the dosage range of 10–30 mg/week. However, when the PGBF dosage exceeded 30 mg/week, the increase in dosage no longer had a significant effect on ALT reduction (Figure 3A). At week 12, there was also a non-linear relationship between PGBF dosage and ALT changes (*p* < 0.0001), which was similar to the relationship observed at week 4 (Figure 3B). Therefore, in the clinical application of PGBF, consideration should be given to the upper limit dosage of 30 mg/week for reducing ALT, and specific dosage adjustments should be made based on individual circumstances.

When the standard dosage was fixed at 10 mg/week, data from only three studies were included for synthesis. In this case, there was a linear relationship between treatment time and ALT changes (*p* = 0.54). As the treatment progressed, ALT levels decreased; however, this decrease was not statistically significant (Figure 3C). When the standard dosage was fixed at 20 mg/week, a non-linear relationship between treatment time and ALT changes was observed (*p* = 0.0012). Before week 20, as treatment time increased, the effect of ALT reduction increased. However, after week 20, ALT levels started to rise, and the significant difference in ALT levels compared to baseline disappeared after week 32 (Figure 3D). Therefore, during the course of PGBF treatment for NASH, consideration should be given to the potential rebound in ALT levels after 20 weeks, and adjustments to medication should be made timely.

### 3.4 Dose–response relationships related to AST

**Figure 4:**
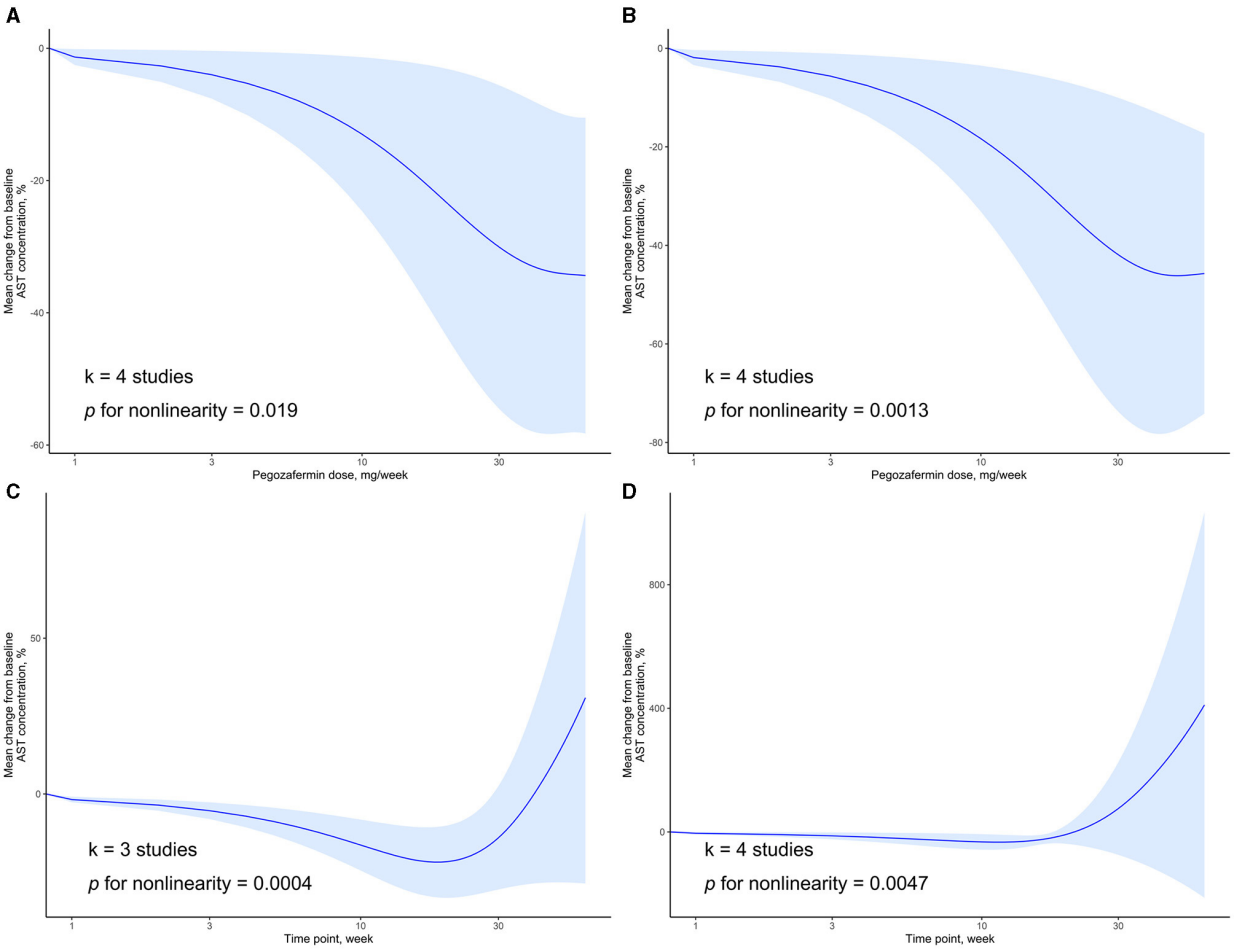
Dose-response relationship of AST change to time point or PGBF dose. **(A)** The nonlinear relationship between AST change and dose at week 4; **(B)** The nonlinear relationship between AST change and dose at week 12; **(C)** The nonlinear relationship between AST change and time point at a dose of 10 mg/week; **(D)** The nonlinear relationship between AST change and time point at a dose of 20 mg/week.

We also examined the dose-response relationship between PGBF dosage, treatment time, and changes in AST levels. At week 4, there was a non-linear relationship between PGBF dosage and AST changes (*p* = 0.019). As the PGBF dosage increased, the effect of AST reduction increased, and the trend was similar to that observed between PGBF dosage and ALT changes. Similar to ALT, when the PGBF dosage exceeded 30 mg/week, the effect of AST reduction was no longer significant (Figure 4A). At week 12, a non-linear relationship between PGBF dosage and AST changes was also observed (*p* = 0.0013), showing a similar trend to that at week 4 (Figure 4B).

When the standard dosage was fixed at 10 mg/week, data from only three studies were included for synthesis. In this case, a non-linear relationship between treatment time and AST changes was observed (*p* = 0.0004). Before week 20, as treatment time increased, the effect of AST reduction increased. However, after week 20, AST levels started to rise, and the significant difference in AST levels compared to baseline disappeared after week 28 (Figure 4C). When the standard dosage was fixed at 20 mg/week, a non-linear relationship between treatment time and AST changes was observed (*p* = 0.0047), showing a similar trend to that observed when the standard dosage was fixed at 10 mg/week (Figure 4D). The changes in AST over time parallel those of ALT and are an important observational indicator in the process of treating NASH with PGBF.

## 4 Discussion

In the dose-response meta-analysis of the four current RCT studies, we found a positive correlation between PGBF dosage and its effect on reducing transaminases. However, a “ceiling effect” was observed when the dosage exceeded 30 mg/week. The term “ceiling effect” refers to the phenomenon where further increases in dosage do not lead to additional improvements in transaminase levels. With a PGBF dosage of 20 mg/week, the average weekly reduction in ALT levels was 8.81%, and AST levels were 6.72% compared to baseline during the first 28 weeks of treatment. However, a rebound effect was observed, and the significance of the difference from baseline diminished.

There are several mechanisms that can explain the improvement of liver function and reduction in transaminases due to PGBF treatment. Firstly, all the studies included in our analysis reported an increase in adiponectin levels with PGBF treatment. Adiponectin is a key adipokine with insulin-sensitizing, anti-inflammatory (31), and anti-fibrotic properties, playing a significant role in regulating lipid metabolism (32). Adiponectin can also antagonize fibrosis by inhibiting the activation of hepatic stellate cells, which play a leading role in fibrosis (33). Therefore, PGBF may improve liver function in NASH patients by promoting an increase in adiponectin levels, leading to a reduction in transaminase levels. Additionally, PGBF has shown beneficial effects on improving lipid profiles in patients (14). Improvement in lipid profiles reflects the enhancement of liver lipid metabolism, reducing hepatic cellular burden and inflammatory responses, thereby lowering transaminase levels (34). Lastly, imaging evaluations have shown that approximately one-third of NASH patients had at least a 15% relative reduction in liver hardness, and a significant decrease in hepatic steatosis was observed after PGBF treatment (14). The reduction in liver hardness and hepatic steatosis indicates an improvement in liver histological status, namely a decrease in liver fibrosis severity. This suggests that PGBF treatment may reduce the occurrence of potential liver complications such as liver failure or liver cancer in NASH patients, thereby improving patients' quality of life and prognosis. Although all four studies included in our analysis observed a reduction in transaminase levels after PGBF treatment, two of the studies did not reach the expected histological endpoints (29, 30). Further research is needed to explore the effects of PGBF on liver histological status, patient body mass index, serum triglycerides, and other indicators.

In the combined results of the random-effects model, there was moderate heterogeneity among the studies on the MD of ALT changes compared to baseline in the highest and lowest PGBF dosage groups at week 12 (*I*^2^ = 64%). The source of this moderate heterogeneity may be the differences in the designs of each study, with variations in the values of the highest and lowest dosages across studies. It is worth mentioning that the other groups showed good homogeneity, with an *I*^2^ statistic of 0%, indicating no significant heterogeneity and making the combined results of the random-effects model highly valuable.

Sensitivity analysis indicated that the differences in the high and low PGBF dosage groups and treatment time in improving transaminase levels would be changed after excluding a certain study. This could be due to the relatively small number of studies and participants included in our analysis, which introduce bias in the sensitivity analysis using the “leave-one-out” method. However, this significant change did not alter the trend of better transaminase reduction with higher PGBF dosage and longer treatment time. In our subsequent exploration of dose-response relationships, we found significant linear or non-linear relationships between PGBF dosage, treatment time, and transaminase reduction. Considering the good homogeneity of our study and the absence of significant publication bias, we still believe that our results are reliably informative.

We found a ceiling effect in PGBF treatment for NASH, indicating that there is a maximum effective dosage of 30 mg/week, and further dosage increase does not provide any additional benefits in terms of transaminase improvement. This could be due to inherent biological limitations in PGBF's mechanism of action or saturation of receptors involved in its effects. Understanding this limitation is crucial for optimizing drug dosing regimens in clinical practice, as higher doses of PGBF may not offer any additional therapeutic advantages but could potentially increase the risk of adverse reactions. This highlights the importance of determining the optimal dosage range that balances efficacy and safety. To better understand the specific reasons behind the observed ceiling effect of PGBF, it is necessary to examine the drug's pharmacokinetics, pharmacodynamics, and potential mechanisms of action in NASH treatment.

We also observed that transaminase levels in NASH patients decreased during the first 20 weeks of PGBF treatment, followed by a rebound with no significant difference from baseline, indicating a complex relationship between NASH response and treatment time. Within the initial 20 weeks, PGBF treatment showed a positive response, with the drug being effective in reducing inflammation or improving liver function in the short term. However, over time, the body may adapt to the drug's effects. Biological systems typically have regulatory mechanisms to maintain internal balance. In response to PGBF treatment, the body may activate compensatory pathways, leading to a rebound in transaminase levels after the initial 20 weeks. Furthermore, long-term administration may cause changes in the liver's response to the drug, resulting in decreased sensitivity to the drug's effects over time, affecting its efficacy. Finally, considering that NASH is a complex progressive disease (2), the therapeutic effect of PGBF may vary with the progression of the disease. It should be noted that only two studies reported follow-up data beyond 18 weeks (29, 30), and further trials are needed to investigate the sustained effects of long-term PGBF treatment.

In the past, some systematic reviews and meta-analyses have attempted to elucidate the role of FGF21 in improving metabolic disorders and liver fibrosis. A meta-analysis conducted by Carbonetti et al. (35) indicated that using FGF21 to treat obese or diabetic patients can significantly improve weight and cholesterol levels, and controlling weight has an effective effect on the liver function status of NASH patients (1), which is basically consistent with the conclusions of our study and suggests that changes in weight are an important intermediate factor affecting the effect of PGBF on transaminases. Regarding the effect of FGF21 on improving liver fibrosis, although the odds ratio obtained by the fixed-effect model of the meta-analysis conducted by Mantovani et al. suggested its benefits in improving liver fibrosis in NASH patients, the significance of this effect disappeared when replaced with a random-effects model (36). Lin et al.'s (37) latest meta-analysis, through the risk ratio obtained by random-effects model, indicated the improvement effect of FGF21 on liver fibrosis and a series of liver function-related indicators including ALT, AST, which is consistent with our findings and inferences. Changes in results after model and statistical indicator replacements may be because the authors only considered the degree of fibrosis improvement at the end of follow-up, ignoring the rebound effect of FGF21 during long-term treatment of NASH, which our study observed. Additionally, other FGF21 analogs besides PGBF may have different effects on improving NASH. A network meta-analysis evaluated the efficacy of PGBF compared to other drugs in treating NASH (38), and PGBF's treatment effect did not show particular advantages over other drugs in NASH treatment. However, this study only included indicators of liver fibrosis improvement, and there were differences in the liver function status of patients included in the studies, making it impossible to comprehensively evaluate the superiority or inferiority of PGBF in improving liver function compared to other drugs. Further consideration is needed for the efficacy of PGBF in combination with multiple drugs for NASH.

Our study has several strengths. Firstly, it is the first dose-response meta-analysis investigating the effect of PGBF on NASH treatment, incorporating innovative statistical methods to quantitatively analyze the impact of PGBF dosage and treatment time on treatment outcomes. Secondly, although our meta-analysis included only four studies, we strictly adhered to rigorous inclusion criteria, incorporating high-quality RCTs with a low risk of bias. Additionally, we applied appropriate statistical methods, including sensitivity analysis, and further explored non-linear relationships using restricted cubic splines, ultimately obtaining robust conclusions, which are of significant reference value for the specific clinical application of PGBF. Thirdly, the included studies showed good homogeneity, providing reliable combined results.

However, our study also has some limitations that need to be acknowledged. Firstly, the participants in our study were geographically limited, involving only 546 participants. Therefore, the robustness and generalizability of PGBF efficacy in other regions globally need further discussion. The limitation of follow-up time in the included studies cannot be ignored, as only two studies reported data beyond 18 weeks, which may not fully reflect the sustainability of treatment effects. This needs further research clarification. Secondly, the included studies may involve various confounding factors, including comorbidities and medical histories of NASH, and residual confounding factors cannot be completely eliminated, which may influence the results. Moreover, our study relied on transaminases as an evaluation indicator of treatment effectiveness. Although this can indirectly assess liver histological status, it may not fully reflect the overall liver function status. Additionally, despite our additional search for gray literature, no additional studies were identified; therefore, there is a possibility that some articles reporting negative results of PGBF or upcoming preprints were overlooked. Finally, our meta-analysis employed summary statistics rather than individual data, and some data in the charts were extracted using relevant software. Although two researchers maintained a double-blind approach during data extraction and resolved related disputes through consensus, it still cannot achieve the precision of research based on individual data.

Our study holds significant clinical implications. As the first meta-analysis to explore the role of PGBF in NASH treatment and the associated dose-response relationship, our findings provide important references for the clinical application of PGBF and the formulation of NASH treatment decisions. For instance, clinicians using PGBF need to monitor the upper limit dosage of 30 mg/week and pay attention to changes in transaminase levels around 20 weeks, adjusting medication promptly to address potential rebounds in transaminase levels. Meanwhile, our study provides data support for conducting new phase clinical trials. Future researchers can expand the study size, extend follow-up time, and conduct PGBF-related NASH treatment studies in other regions worldwide to explore its effects on different populations. Additionally, to better assess PGBF's role in improving liver function, further discussions are needed on other relevant indicators such as liver fat content, serum triglycerides, and others.

## 5 Conclusions

In summary, our study reveals a positive non-linear dose-response relationship between PGBF dosage and the reduction of transaminases in NASH patients. We found a maximum effective dosage of 30 mg/week, beyond which further dosage increase does not lead to additional benefits in improving transaminases. During PGBF treatment, NASH patients experienced a significant decrease in transaminase levels in the first 20 weeks, followed by a rebound phenomenon. This information can provide valuable guidance for clinicians in developing specific dosing strategies for PGBF in NASH treatment.

## Data availability statement

The raw data supporting the conclusions of this article will be made available by the authors, without undue reservation.

## Author contributions

YL: Conceptualization, Data curation, Formal analysis, Investigation, Methodology, Project administration, Resources, Supervision, Validation, Visualization, Writing – original draft, Writing – review & editing. BY: Data curation, Formal analysis, Writing – review & editing. YB: Data curation, Writing – review & editing. JL: Data curation, Writing – review & editing. YJ: Conceptualization, Investigation, Resources, Supervision, Visualization, Writing – review & editing.
