# Supplementary material for: Pegbelfermin for reducing transaminase levels in patients with non-alcoholic steatohepatitis: a dose-response meta-analysis of randomized controlled trials
Source: Front Med (Lausanne). 2024 Apr 5;11:1293336. doi: 10.3389/fmed.2024.1293336 (PMC11026620; doi:10.3389/fmed.2024.1293336)
Supplement: Supplementary file 2 [file Table_2.DOCX]

**Date 2023/07/18**

***PubMed***

#1 "fibroblast growth factor 21"[Supplementary Concept] OR "fgf21 protein human"[Supplementary Concept] OR "Pegbelfermin"[Supplementary Concept]

#2 "Pegbelfermin"[Title/Abstract] OR "BMS-986036"[Title/Abstract] OR " fibroblast growth factor 21*"[Title/Abstract] OR " FGF21*"[Title/Abstract]

#3 #1 OR #2

#4 "non alcoholic fatty liver disease" [MeSH Terms]

#5 "non alcoholic fatty liver disease"[Title/Abstract] OR "non alcoholic fatty liver disease"[Title/Abstract] OR "NAFLD"[Title/Abstract] OR "Nonalcoholic Fatty Liver Disease"[Title/Abstract] OR "fatty liver nonalcoholic"[Title/Abstract] OR "liver nonalcoholic fatty"[Title/Abstract] OR "nonalcoholic fatty*"[Title/Abstract] OR "nonalcoholic steatohepatiti*"[Title/Abstract] OR "steatohepatitis nonalcoholic"[Title/Abstract]

#6 #4 OR #5

#7 #3 AND #6

***Embase***

#1 'fibroblast growth factor 21'/exp

#2 'Pegbelfermin':ti,ab,kw OR 'BMS-986036':ti,ab,kw OR ' fibroblast growth factor 21*':ti,ab,kw OR ' FGF21*':ti,ab,kw

#3 #1 OR #2

#4 'nonalcoholic steatohepatitis'/exp

#5 'non-alcoholic fatty liver disease':ti,ab,kw OR 'non alcoholic fatty liver disease':ti,ab,kw OR nafld:ti,ab,kw OR 'nonalcoholic fatty liver disease':ti,ab,kw OR 'fatty liver, nonalcoholic':ti,ab,kw OR 'fatty livers, nonalcoholic':ti,ab,kw OR 'liver, nonalcoholic fatty':ti,ab,kw OR 'livers, nonalcoholic fatty':ti,ab,kw OR 'nonalcoholic fatty*':ti,ab,kw OR 'nonalcoholic steatohepatiti*':ti,ab,kw OR 'steatohepatitides, nonalcoholic':ti,ab,kw OR 'steatohepatitis, nonalcoholic':ti,ab,kw

#6 #4 OR #5

#7 #3 AND #6

***Cochrane Library***

#1 ("Pegbelfermin" OR "BMS-986036" OR "fibroblast growth factor 21" OR "FGF21"):ti,ab,kw

#2 MeSH descriptor: [non alcoholic fatty liver disease] explode all trees

#3 ("Non-alcoholic Fatty Liver Disease" OR "Non alcoholic Fatty Liver Disease" OR NAFLD OR "Nonalcoholic Fatty Liver Disease" OR "Fatty Liver, Nonalcoholic" OR "Fatty Livers, Nonalcoholic" OR "Liver, Nonalcoholic Fatty" OR "Livers, Nonalcoholic Fatty" OR "Nonalcoholic Fatty*" OR "Nonalcoholic Steatohepatiti*" OR "Steatohepatitides, Nonalcoholic" OR "Steatohepatitis, Nonalcoholic"):ti,ab,kw

#4 #2 OR #3

#5 #1 AND #4

***Web of Science***

#1 TS=("Pegbelfermin" OR "BMS-986036" OR "fibroblast growth factor 21" OR "FGF21"):ti,ab,kw

#2 TS=("Non-alcoholic Fatty Liver Disease" OR "Non alcoholic Fatty Liver Disease" OR NAFLD OR "Nonalcoholic Fatty Liver Disease" OR "Fatty Liver, Nonalcoholic" OR "Fatty Livers, Nonalcoholic" OR "Liver, Nonalcoholic Fatty" OR "Livers, Nonalcoholic Fatty" OR "Nonalcoholic Fatty*" OR "Nonalcoholic Steatohepatiti*" OR "Steatohepatitides, Nonalcoholic" OR "Steatohepatitis, Nonalcoholic")

#3 #1 AND #2
